# A novel method for automatically analysing the activity of fast-moving animals: a case study of *Callimico goeldii* monkeys housed in a zoological garden

**DOI:** 10.1038/s41598-023-38472-4

**Published:** 2023-07-16

**Authors:** Zenon Nieckarz, Jacek Nowicki, Karolina Labocha, Krzysztof Pawlak

**Affiliations:** 1 Department of Experimental Computer Physics, Institute of Physics, Jagiellonian University in Cracow, Cracow, Poland; 2 Department of Genetics, Animal Breeding and Ethology, University of Agriculture in Cracow, Cracow, Poland; 3 Silesian Zoological Garden, Chorzów, Poland; 4 Department of Zoology and Animal Welfare, University of Agriculture in Cracow, Aleja Adama Mickiewicza 24/28, 30-059 Kraków, Poland

**Keywords:** Biological techniques, Ecology, Psychology, Zoology

## Abstract

Behavioural indices are recognised as important criteria for assessing animal welfare. One of the basic animal behaviours included in ethograms is their activity. The assessment of fast-moving animals, performed by humans using the visual observation method, is difficult and not very objective. Therefore, the aim of the research was to develop a method of automated analysis of animal activity, particularly useful in the observation of quick and lively individuals, and to prove its suitability for assessing the behaviour of fast-moving animals. A method of automatically assessing animal activity was developed using digital image analysis, with the Python programming language and the OpenCV library being the foundational tools. The research model was *Callimico goeldii* monkeys housed in a zoological garden. This method has been proved to correlate well (Rs = 0.76) with the visual method of animal behaviour analysis. The developed automatic evaluation of animal behaviour is many times faster than visual analysis, and it enables precise assessment of the daily activity of fast-moving groups of animals. The use of this system makes it possible to obtain an activity index with sub-second resolution, which allows it to be used in online mode as a detector of abnormal animal activity, e.g. early detection of illnesses or sudden events that are manifested by increased or decreased activity in relation to the standard activity pattern.

## Introduction

Behavioural indices are recognised as important criteria for assessing animal welfare. There are several divisions of methods of observing and recording animal behaviour. Originally, only direct methods with the presence of an observer at the animal’s location were used^1^. Manual analysis of behaviour is massively limited by two insurmountable obstacles: the complexity of the behavioural patterns and human bias. Disorders in animal behaviour resulting from the presence of an observer in their vicinity and the development of technology have led to the increased use of indirect methods involving remote recording of behaviour. Initially, analogue recordings were made on film tape and later on, magnetic tapes (VHS cassettes) were used, with the help of time-lapse video recorders. Nowadays, security cameras and digital recording on flash media (HDD, SSD, memory cards, etc.) are most often used in observing behaviour and gathering data. The prevalence of security cameras, combined with their good image quality and low prices, has led to the increased use of images as a source of information about animal activity. This thesis is confirmed by the growing number of scientific papers in which video recording was used as a source of information^2,3^. In addition to the most commonly used animal observation techniques involving video monitoring, other methods of recording animal behaviour are also being applied, such as Radio Frequency Identification (RFID)–most frequently in enclosed spaces^4^ and the Global Positioning System (GPS) in the case of animals living in the wild or kept in open spaces^5^.

Due to the presence in zoological gardens of numerous animal species, whose biology and behaviour are still poorly understood, it is important to observe and analyse their behaviour to ensure an adequate level of welfare.

One of the species found in large numbers in various zoological gardens is *Callimico goeldii (*called *Callimico*, mico or Goeldi’ monkeys in further parts of this paper*)* which is a South American primate found in the tropical rain forests of the upper Amazon region^6^. *Callimico* was first described in 1904 by Oldfield Thomas and named after the Swiss zoologist Emil Goeldi^7^. The body of this animal measures from 19 to 25 cm, the tail from 26 to 35 cm, and their body weight is about 360 g. In captivity, body weight is usually higher, around 500 g. It is typical for this species to give birth to only one young per litter. Black miko breed twice a year. Births occur from February to March, and at the end of August. Black mikos in nature live in small groups (on average about 4–5 adults per group, minimum 2 and maximum 12)^8^.

### Behaviour of *Callimico goeldii*

In nature, *Callimico goeldii* migrates and feeds in the low parts of the forest, preferring, like most arboreal animals, dense thickets. It lives in primary forests with dense undergrowth, as well as in secondary forests, but it thrives particularly well in bamboo forests. *Callimico* is considered a "specialist in the undergrowth"–it lives at a height of 4–5 m above the forest undergrowth^9^. It has many anatomical adaptations for vertically grasping small trees and jumping between them, and for grasping large tree trunks and lianas at low heights. It has long hind limbs and modified arm bones to dissipate the forces of long jumps. In addition, they have special claws thanks to which they cling to large vertical and sharply inclined supports in the forest understory^10,11^. *Callimico* has been observed to accelerate rapidly over short distances^12^ using quadrupedal running: a rapid form of diagonal-sequence/diagonal-couplet pronograde travel that does not include an in-air phase of stride. Goeldi’s monkeys are characterised by rapid and frequent changes of location, positional behaviour, and substrate. In nature, *Callimico* feeds mainly on fruits, mushrooms and small animals. Groups of *Callimico goeldii* usually forage in the low level of the forest, between thin twigs, and on litter^8^. Callitrichidae's hunting strategy includes: scanning, locomotion, pouncing and capturing the prey either with the mouth or hands^13^^.^.

As for their daily activity in nature, Callimicos go to their resting place between 17:00 and 18:00 and stay there until 06:00. The sleeping place is a typical tangle of branches, lianas, vines, and dense vegetation at a height of about 10 m from the forest floor. After long-term observations in northwest Bolivia, their overall activity is as follows: resting 66%, travelling (movement) 17%, eating 9%, searching for food 6%, other behaviour 2%. *Callimico* spends more time awake while resting (as much as 87%) than other tamarins^8^.

One of the basic animal behaviours included in ethograms is mobility, which can be measured by an animal's level of activity in a given time period. Movement of an organism, defined as a change in the spatial location of the whole individual in time, is a fundamental characteristic of life, driven by processes that act across multiple spatial and temporal scales^14^. Animal movement is an essential characteristic of biological systems, shaping their structure and dynamics from individual behaviour to the community or ecosystem level^15^. Animal activity can be affected by, among other things, the behaviour of other individuals, health, the desire to satisfy hunger, to find a partner or by environmental conditions where the animals are kept^14^. So it can be stated that assessing animal behaviour is essential to understanding animal life^16^.

The specificity of the behaviour of *Callimico goeldii* and other agile and fast-moving animals makes it difficult and time consuming to visually observe and analyse their behaviour^3^. As it is known, efforts are being made to minimise the differences between natural behavioural patterns observed in the wild and animal behaviours in zoos. Monitoring animals in zoos helps to identify the extent to which their behaviour deviates from natural patterns. Consequently, the analysis of behaviours in zoos should be easy and quick to perform.

Various methods of automatic monitoring of animal activity can be found in the literature. These methods are based on different algorithms and computer tools, with the most popular ones being: fitting to a defined model^17,18^, reference image^19,20^, intensity difference^21^, neural networks^22,23^, support vector machine^18^.

It should be noted that the methods described in the literature require specific technical conditions that may not be met in zoos or require advanced computations (e.g., algorithm training processes), which prolongs the time required for the data processing and, in extreme cases, makes it impossible to use average equipment.

Therefore, the aim of the research was to develop a method of automated analysis of animal activity that would be particularly useful in the observation of agile individuals and to prove its suitability for assessing the behaviour of fast-moving animals kept in zoo, such as Goeldi’s monkeys.

## Material and methods

### Ethical approval

Our research (permit no. 29/2015) consisted of non-invasive, camera-based observation of animals, without introducing any additional factors into their environment.

Such activities, in accordance with DIRECTIVE 2010/63/EU OF THE EUROPEAN PARLIAMENT AND OF THE COUNCIL of 22 September 2010 on the protection of animals used for scientific purposes, do not require the consent of the ethics committee.

### The research object

Video observations were carried out in the Silesian Zoological Garden in Chorzów, Poland in enclosures that house Goeldi’s monkeys (*Callimico goeldii*).

We collected behavioural data for one particular group of animals – a family of Goeldi’s monkeys (*Callimico goeldii*). The family group consisted of nine animals: five females and four males.

### Housing

The indoor enclosure for *Callimico goeldii* was located in the monkey house pavilion at the Silesian Zoological Garden in Chorzów. The dimensions of the indoor enclosure for these monkeys were 4 m × 2.85 m. From the audience side and the run, the room is glazed. The rest of the walls are made of decorative elements imitating stones. The temperature in the room was maintained at 24 °C. The humidity in the room was about 75% in order to bring the climatic conditions closer to those that occur in nature. The humidity was controlled daily and, if necessary, increased by manual sprinkling of the substrate on the floor. There is pine bark on the substrate to keep it moist. In addition to permanently installed elements, such as the wooden shelves, there were also four heat emitting radiators in the room, enclosed in a metal mesh for security purposes. The room has thin and thick branches that are features of the decor, and shelves on which animals can walk and jump. Additionally, small coniferous and deciduous trees harvested from the garden are placed in the enclosure on a seasonal basis. The caretakers tried to ensure that the room had a lot of live plants that help maintain adequate humidity.

### Measuring equipment and gathered data

In the indoor enclosures of the Silesian Zoological Garden in Chorzów, a family of Goeldi’s monkeys was observed for 12 days (24 h observation) from 3 to 14 March using a digital camera (Model: BCS AT V554OSDIR48), System PAL, CCD 1/3 SONY. This camera was installed in the upper parts of the room, at a height of 3.2 m. It was aimed at the shelves where the monkeys most often stayed and interacted with each other.

The collected footage covers 12 days, i.e. 288 h of recording. The video was recorded with a BCS recorder (BCS-0804LE-L) equipped with a 1 TB SATA HDD and a DVD recorder. Transmission of video was performed through 75 Ohm Conotech NS100 Trishield Coaxial Cable. The camera was connected to the above-mentioned recorder with a BNC connector. A total of 14,515,200 frames were recorded during the analysed period. The image from the camera was recorded at a resolution of 704 × 576 (W×H) pixels and frame per second rate of 14 Hz. The camera was equipped with additional infrared lighting, which made it possible to observe the animals at night as well.

BCS Smart Player (ver. 3.44.0) software was utilised for the manual visual assessment of animal activity. Automated numerical procedures were developed in the Python programming environment (ver. 3.8) and using pre-built CPU-only OpenCV packages (opencv-python, MIT Licence). Calculations and automated analyses were performed on a computer with average technical parameters (Intel(R) Xeon(R) CPU E3-1240 v3 @ 3.40 GHz; RAM 16 GB; NVIDIA Quadro K2000 graphics) running Windows 10 (× 64).

### Behaviour analysis of Goeldi’s monkeys performed by an observer

The visual analysis of Goeldi’s monkey behaviour by a human observer, based on the collected video material, was performed in two ways:

- *General analysis*–analysis of the rhythm of the round-the-clock phase of activity and resting periods of the animals based on the total video recording material collected (duration of analysis—72 h)
- *Detailed analysis*–analysis of activity during the daytime based on a 10 min, randomly selected fragment of video recordings according to the scale presented below (M index):

0–a maximum of two individuals show physical activity (no movement in the space, visible single movements of body parts).

1–three and more individuals show physical activity (no movement in the space, visible single movements of body parts).

2–a maximum of four individuals move in the space.

3–a minimum of five individuals move in the space.

In the case where the animals moved within the space, no attention was paid to the movement of animals not moving away from their place. Such activity was given at least two points.

This analysis took the observer approximately 95 min to complete.

The proposed scale of detailed analysis refers to the methodology of automatic image processing described below, where the body movement without the movement of the individual in the space caused a change in a smaller number of pixels compared to when the monkeys moved in the space.

### The automatic method of activity assessment for very fast-moving animals

All collected recordings (288 h-long files–analysis time less than 60 min) were used for the automatic analysis of *Callimico*’s behaviour. The results obtained in the detailed analysis were compared to the results obtained in the automatic analysis.

The method for the automatic assessment of animal activity presented below has been developed and optimised for Goeldi’s monkeys (*Callimico goeldii*) and takes into account, among other things, the herd way of life, the animals’ movement speed and the low contrast of animals against the background. Automated open field analyses of a video tracking system that analyses animal behaviour may provide a more sensitive analysis of the animals’ behaviour than that made by the human observer.

**Figure 1:**
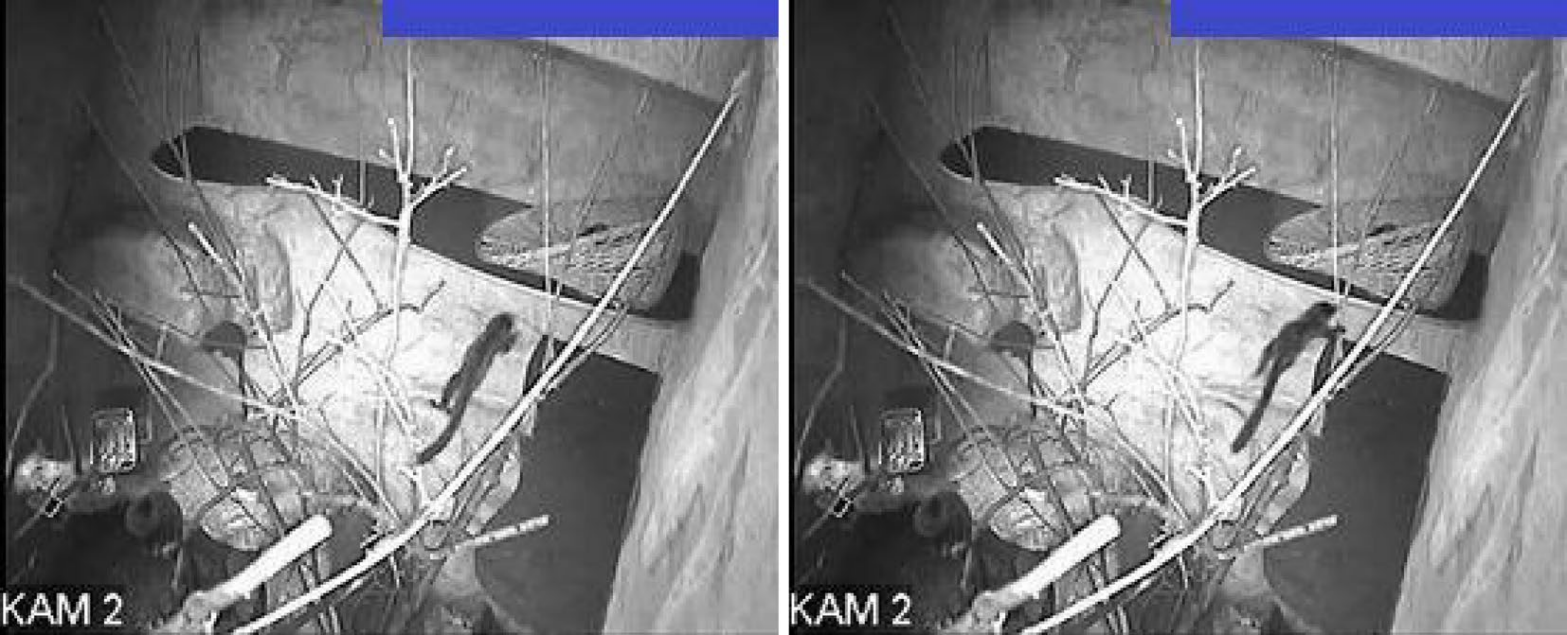
Examples of two image frames of film recorded one after the other (current—panel Left, next—panel Right)**,** the images were recorded in the daytime. (KAM2, FPS = 14, Δt = 0.071 s). The blue rectangular areas mask the areas on the images where the date and time of registration is displayed.

Figure 1 presents an example of two image frames recorded one by one (FPS = 14, Δt = 71.4 ms). It is clearly seen that the animal in the centre of the image has a different body position. This shows how fast these animals can move. At the same time, this behaviour makes it difficult to assess the activity manually.

In general, the proposed automatic activity evaluation method described below is based on an algorithm that calculates the difference between the i-th current image frame and the information contained in N image frames before the current i-th image. As a result of the algorithm, the A_i_ index values were obtained for every image frame (except for the first N frames).

In detail, daily periods were captured in colour as a movie, whereas night periods were captured in grayscale. Thus, in the initial stage of analysis, each frame was converted to grayscale, with values ranging from 0 (black) to 255 (white). This ensured that daily and nightly observations were subjected to the same algorithmic analysis. Each i-th image was compared with a set of N = 28 image frames (period equal to 2 s) before the analysed image. For each pixel in the analysed image, the squared Mahalanobis distance (SMD)^24^ was calculated in grayscale between the given pixel and 28 pixels forming the background grayscale distribution model. This was done to determine whether a given pixel is well described by the model. Next, if the SMD was below value 20, then the pixel was well described by the model and no change in the image was observed; in other cases, changes have been detected. The value of 20 was determined experimentally as a compromise between the sensitivity of motion detection and the probability of generating a false signals.

Finally, a binary mask of the image was constructed that presents changes between the current image and the 28-th background images (immediately preceding the i-th analysed), which represented movement areas. Using the Python language^25^ and the OpenCV library ^26^ , the calculations were performed using the following instructions:

*Line 1: MOG* = *cv2.createBackgroundSubtractorMOG2(history* = *28, varThreshold* = *20, detectShadows* = *False);*

*Line 2: kernel* = *cv2.getStructuringElement(cv2.MORPH_ELLIPSE,(5,5))*

*Line 3: mask_result* = *MOG.apply(frame_current);*

*Line 4: eroded* = *cv2.erode(mask_frame, kernel);*

Upon execution of the Python script, a dedicated object *MOG* is created on the first instruction with the parameters set accordingly; where: *history* is the number of frames in the background model; *varThreshold* is the threshold value of the squared Mahalanobis distance; and *detected Shadows* is a logical variable that indicates whether detected changes between image and background will be indicated as binary only or also intermediate values. This line of code is only executed once.

As a result of the execution of the second line of code, the object kernel is created as a structural element for the erosion morphological filter (shape: ellipse, size: 5 × 5). This line of code is also executed once.

In the third line of code, we obtained a mask image *(mask_result)* where the changes in pixels are represented by white pixels, while black pixels represent the unchanged areas. *frame_current* represents the *i-th* current image frame taken from the video.

Next to the binary mask which represented movement, the erosion filter was applied^27^. The kernel size of the filter was used as the ellipse element (5 × 5) for cleaning noise in the binary mask image. Noise can be produced by the image sensor (camera) as well as the software conversion and compression process.

**Figure 2:**
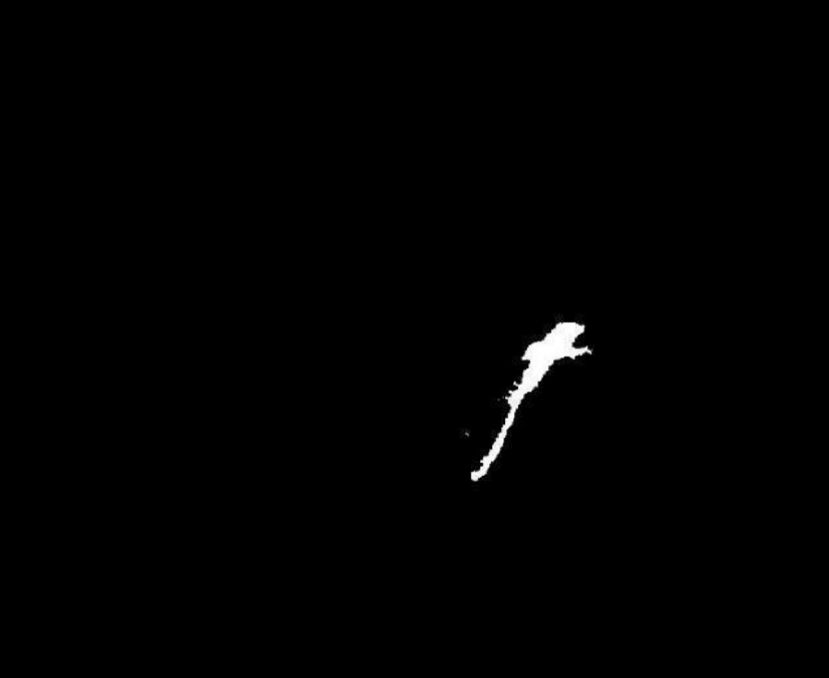
The final image of the difference between the images presented in Fig. 1 (panel Right) and the background model image created based on the previous 28 image frames. White pixels indicate areas where changes were detected.

Figure 2 shows the final mask image depicting the difference between the image presented in Fig. 1 (panel Right) and the background model image generated using the preceding 28 image frames.

Creating a dynamic background from the 28 images preceding the current analysed image allows for the neutralisation of the impact of changes in the scenery in the view of camera (changes in the position of objects) resulting from the actions of animals and caretakers (food containers).

For each mask image after filtration, the fraction of white pixels in the whole mask image (called the *A* index) was calculated, which is proportional to the activity of the animals observed by the camera. In fact, the *A* index corresponds to animal activity, containing elements such as the speed and number of animal movements.

### Statistical analysis

A statistical analysis was performed using the STATISTICA software system (ver. 13.3, TIBCO Software Inc.). All data in the Table 1 are reported as mean values ± SEM.

**Table 1:** Average daily (6:30–18:30) values of A0 and A1 indices, along with their standard error of the mean, determined for 12 days (from 3 March 2016 to 14 March 2016).

Date–time period	A0 index mean (± SEM)	A1 index mean (± SEM)
03.03.2016—6:30–18:30	0.12 (± 0.06)	0.08 (± 0.04)
04.03.2016—6:30–18:30	0.16 (± 0.08)	0.11 (± 0.07)
05.03.2016—6:30–18:30	0.15 (± 0.08)	0.10 (± 0.06)
06.03.2016—6:30–18:30	0.16 (± 0.08)	0.11 (± 0.06)
07.03.2016—6:30–18:30	0.13 (± 0.06)	0.08 (± 0.05)
08.03.2016—6:30–18:30	0.16 (± 0.10)	0.11 (± 0.08)
09.03.2016—6:30–18:30	0.14 (± 0.07)	0.09 (± 0.05)
10.03.2016—6:30–18:30	0.13 (± 0.06)	0.09 (± 0.04)
11.03.2016—6:30–18:30	0.15 (± 0.10)	0.10 (± 0.08)
12.03.2016—6:30–18:30	0.15 (± 0.05)	0.10 (± 0.03)
13.03.2016—6:30–18:30	0.15 (± 0.07)	0.10 (± 0.05)
14.03.2016—6:30–18:30	0.14 (± 0.07)	0.09 (± 0.05)

The normality of the data was tested by means of the Kolmogorov–Smirnov test. In all tested groups, the distributions were significantly different from the normal. Spearman’s rank correlation (Rs) analysis was performed to study the relation between analysed indices.

The Kruskal–Wallis ANOVA rank test examined whether there was a difference between the A index grouped by the independent variable (M index). In order to check which groups of the A index are statistically significant, multiple comparisons were carried out using the post-hoc Dunn’s Test.

## Results and discussion

The behaviour of animals, and in particular their activity, is an important indicator of animal welfare.

The first step in assessing the monkeys’ activity was a visual *general analysis* of the collected video material. The collected observations have enabled us to determine a distinct rhythmicity in the activity and rest phases of Goeldi’s monkeys depending on the time of day. In addition, the activity of these animals was affected by the actions performed by the staff, e.g. feeding or cleaning the enclosures. The beginning of the 24 h period of the monkeys’ activity took place between 6:00 and 6:30 and ended between 18:30 and 19:00. During the daylight hours, activity peaks were observed recurring at specific hours. This was consistent on subsequent observation days. Daylight activity of *Callimico goeldi* is typical in natural conditions as well^8^.

Automating and therefore objectifying the determination of animal activity is important, especially in zoological gardens where animals are deprived of their natural habitat. The automatic method described in this paper allows for quick and objective measurements of the activity of fast-moving animals. In addition, the proposed method allows for an assessment of animal behaviour not only moving in one plane (e.g. on the floor) but also in three-dimensional space.

### Comparison of two independent methods (automatic and visual)

**Figure 3:**
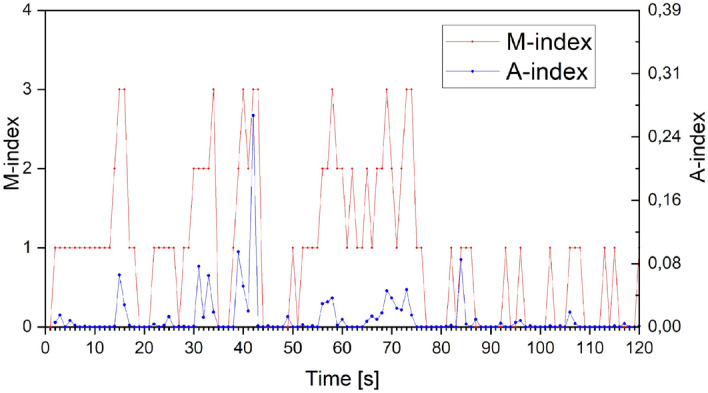
Time domain distribution (120 s, from 11:00 to 11:02 LT, 04.03.2016) of indices *M* (red colour) and *A* (blue colour). *M*-index—animal activity index according to the scale described earlier (see detailed analysis in methods); *A*-index calculated automatically, described earlier in the text.

Figure 3 presents the time distribution (120 s with a resolution of 1 s) for the *M* and *A* indices, as an example of a convenient optically comparison of characteristics of both graphs. It is evident that for M > 1, the A index also assumes values distinctly different from zero.

**Figure 4:**
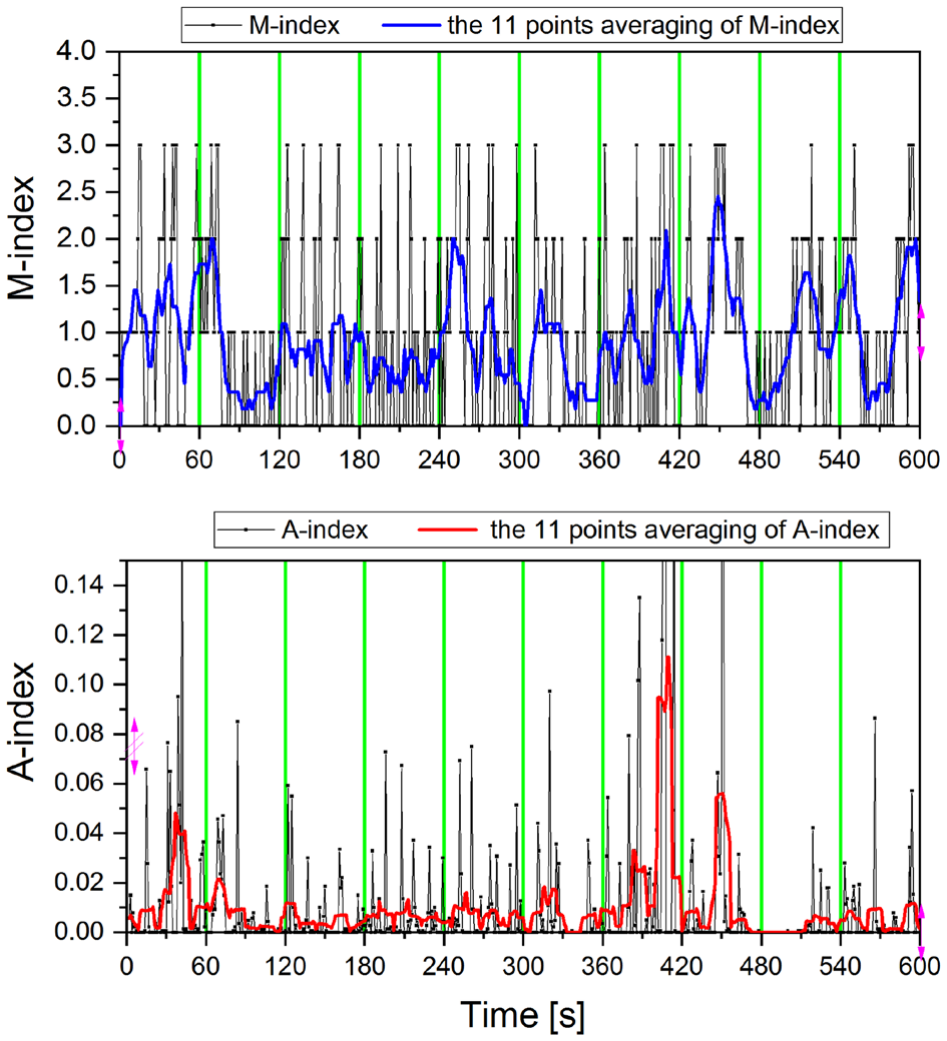
The graph shows the second indices M (top panel) and A (bottom panel) and their 11 averaging points. Presented data cover the period 600 s (from 11:00 to 11:10 LT, 04.03.2016).

In Fig. 4, the time domain of the M index (top panel) and A index (bottom panel) were calculated for a 10 min period (from 11:00 to 11:10 LT, 04.03.2016), and the 11 s simple moving average curve was displayed. The 11 s width of the moving average was chosen experimentally, taking into account the visual clarity of the chart. By analysing the shape of the moving average charts calculated for both indices, a clear difference in the dynamics of the two indicators can be observed.

It has to be noted that index M can only take four discrete levels (0, 1, 2, 3) and index A can take values from 0 to 100 with a resolution of 0.01. Consequently, with a good approximation, it can be concluded that index A is almost continuous. The consequences of the difference in distributions of both indicators can be clearly seen in Figs. 3 and 4. Neither of the indices (M and A) form a normal distribution, and the Spearman correlation coefficient (Rs) between them is 0.49 and is statistically significant (*p* < 0.05).

The correlation between A1 and M2 (0.76) is significantly lower than that achieved in the study conducted by Ott et al.^28^ on pigs, where Rs values of approximately 0.9 were obtained between the automatic and manual methods. It should also be emphasised that the speed of movement of Goeldi’s monkeys is much higher than that of pigs while the body size of these animals is smaller than that of pigs. Such characteristic movement of monkeys makes manual observation difficult and undoubtedly affects the obtained correlation value.

**Figure 5:**
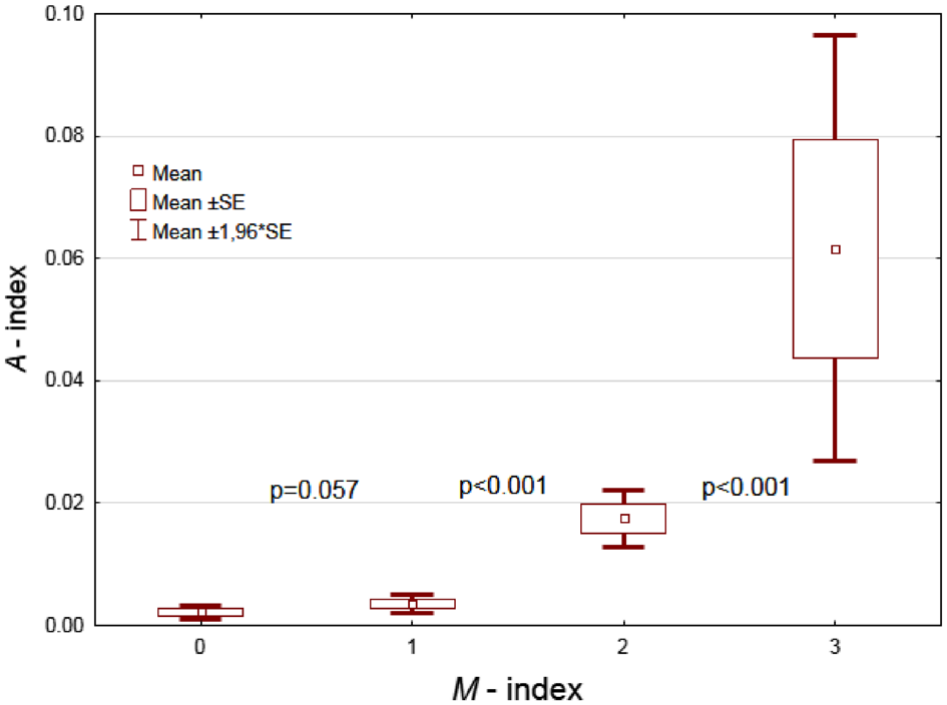
The results of post-hoc Dunn’s Test.

The Kruskal–Wallis rank ANOVA test showed that the A index (dependent variable) divided into four groups according to the value of the M index (independent variable) demonstrates that at least one group of A indices in the four groups differs from the others (*p* < 0.0001). The analysis of multiple comparisons of rank means (post-hoc Dunn’s Test) for all discrete values of the M index showed that there is only no statistically significant difference between the median of the A indices grouped by M = 0 and M = 1 (*p* = 0.057), while the other groups differ significantly *p* < 0.001 (Fig. 5).

Based on the M index time data set, the three time-activity indices, namely M0, M1 and M2, were calculated for every minute. Specifically, M0 represents the fraction of time during which the M index was greater than 0, while M1 represents the fraction of time during which the M index was greater than 1. Lastly M2 denotes the fraction of time during which the M index was greater than 2. Based on the A index, the activity indices A0 and A1 were calculated for every minute, representing the fraction of time when the A index was greater than 0 and greater than 0.01 respectively.

The highest and most significant correlation coefficient–0.76–was found between indices A1 and M2 (*p* = 0.010). Additionally the A1 mean value (0.111) is closer to the M2 mean value (0.077) but significantly different at *p* = 0.05. It should be noted that the M2 indicator provides information about a large number of individuals moving in the observed space. In the automated method, a large number of moving animals results in a large number of white pixels (in the mask image) indicating areas where changes have been detected. As a consequence, this raises the value of the A indicator above the adopted threshold of 0.01, creating a non-zero value of the A1 indicator. This means that the M2 indicator determined by the time-consuming visual method can be replaced by the A1 indicator determined easily and quickly by the automatic method.

**Figure 6:**
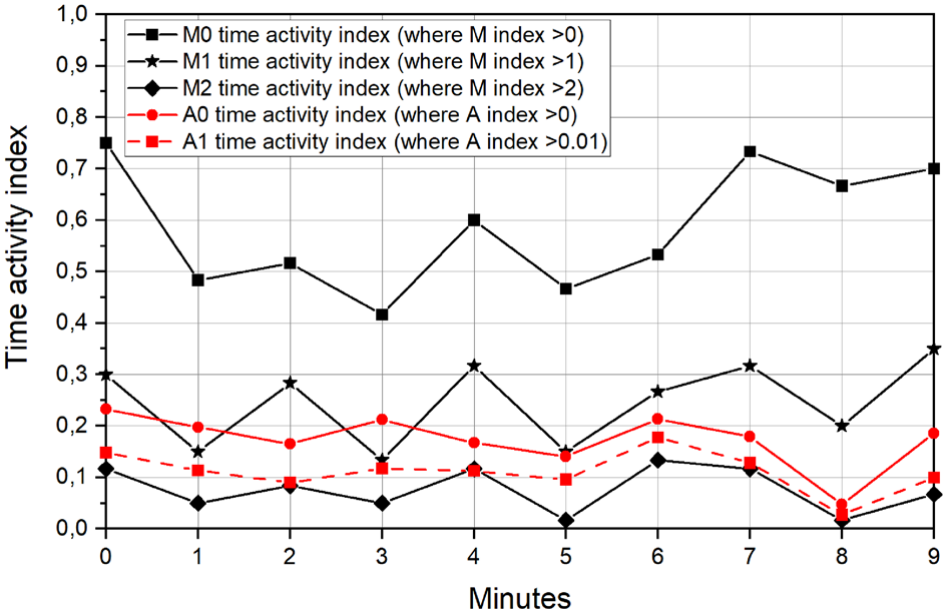
The time-activity indexes calculated using the human method of analysis (M0, M1, M2) and the automatic method (A0, A1).

The next highest correlation coefficient – 0.60 – was found between indices A0 and M2 (p = 0.064). The A0 mean value (0.174) is the nearest to the M1 mean value (0.247) but still significantly different at p = 0.05 (Fig. 6) .

### Long term activity monitoring by automated method

**Figure 7:**
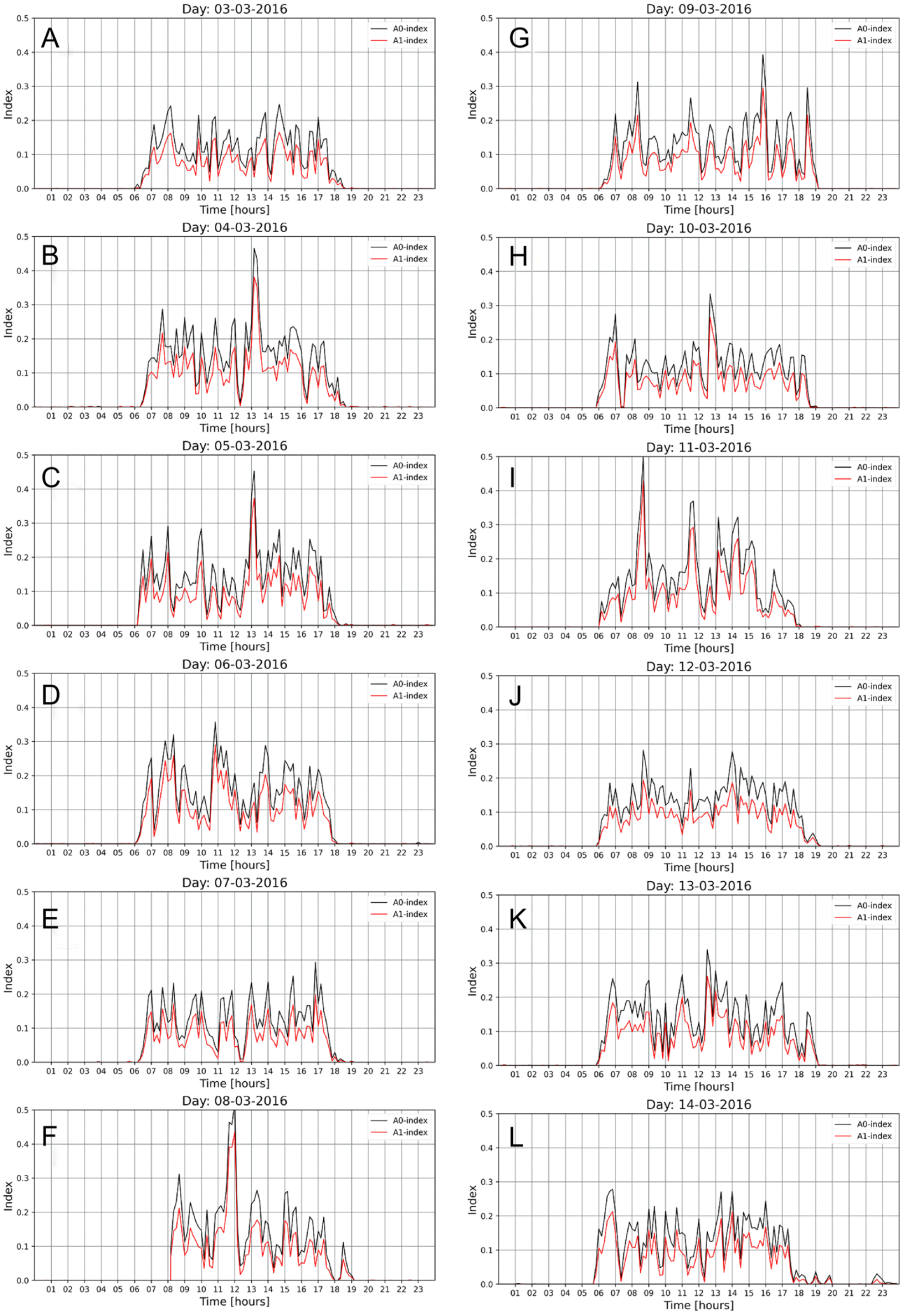
Time distribution of the A0 and A1 activity indexes during 12 days with the 10 min of resolution.

Figure 7 illustrates the trend of the A0 and A1 indices, which describe the animals activity with a resolution of 10 min in the examined period of 12 24 h periods (03–14.03.2016). During the analysed 24 h periods, in the daytime (between 6:30 and 18:30), the A0 index value ranges from 0.05 to 0.30. The exception is the repeated peaks (on 4 and 5 March) in both indices occurring between 13:00 and 14:00. The reason for such high animal activity in the peak periods is the presence of a guardian (feeding, cleaning work), which was confirmed by visual analysis of the video recordings at the time indicated by the A0 and A1 indices. On the other hand, at nighttime (from 19:00 to 6:00) the A0 and A1 indices were both zero, except for a few cases in which both indices reached maximum values below 0.01 (panels A–K).

The activity of Goeldi’s monkeys shown in Fig. 7 corresponds to the results of the assessment conducted by the *general analysis* method and is characterised by a clear rhythmicity depending on the time of a day. Showing activity at certain times of the day (from approx. 6:00 to 19:00) correlates with the lighting program used in the zoological garden, which is in accordance with the recommendations contained in the Callimico Species Survival Plan Husbandry Manual^29^. The activity peaks visible in the charts are related to the distribution of food, which was confirmed during the *general analysis*. Other authors also show an increase in the activity of animals related to feeding behaviour^30^. Studies conducted on capuchin monkeys have shown that observing their group members eating food increases the activity of other animals^31^, which is consistent with our own observations, when the feeding activity of one individual increased the interest of other animals, and then they began to show eating behaviour.

In the analysed period, the average values of the A0 and A1 indices during daylight hours ranged from 0.124 to 0.163 for A0 and from 0.082 to 0.113 for A1 (Table 1).

Data of this type, as listed in Table 1, allow a quantitative, in the long-term (e.g. one year), demonstration of changes in the animals’ activity, e.g. under the influence of weather conditions, or emerging disorders in animal welfare.

The usefulness of this automatic animal activity assessment system was also confirmed by the A0 and A1 indices on day 14 (Fig. 7 panel L), which were non-zero from 19:00 indicating higher activity compared to all previous days. The data obtained from the *general analysis* of the video showed that from about 19:00 one of the females displayed unusual behaviour and was moving around in circles, was nervous, turned around a lot and was restless. It was at this point that the birthing process began. The monkey then rested with the group until 22:40, when it was observed to move to the shelf located under the radiator bar, which was shown by an increase in activity (Fig. 7, Panel L).

In the literature, methods based on animal detection from video frames are most often used for the individual assessment of the behaviour of animals in cages, e.g. hens^32^, red foxes^33^, pigs in a pen^28,34,35^, monkeys^36,37^, and drosophila^38^. However, these methods introduce initial conditions, e.g. they require the following: the presence of only one object in the cage^32^; a model, a sample of training data, and a method training process^34,37^; the need to have the camera pointed perpendicular to the floor to capture the entire pen^28,35^.

Meanwhile, the analysis method presented in this paper (a) is based on free software, (b) does not introduce any initial conditions (technical, hardware), (c) does not require a learning/training process, and (d) does not require the introduction of model assumptions about the observed objects. Changes in the activity of animals can be detected quickly which enables the reactions of animal handlers and finding the factor disturbing the typical daily activity of animals. For example, an increase or decrease in activity may indicate the existence of a disease that is initially difficult to detect.e.g. pasteurella disease. Moreover, the presented method detects unusual changes in activity, thanks to which, for example, zoo employees can intervene to identify the causes. An example is the detection of activity changes during labour. The system can therefore act as an indicator of important events relevant to the welfare of animals in the zoo.

In the paper, we have presented the basic possibilities of using the A index (division by day/night), and in future applications, smaller intervals can be introduced to target the detection/monitoring of other expected specific (or non-specific) animal behaviours.

## Conclusion

Summing up, it can be concluded that the method described herein is suitable for the automatic assessment of the activity level of fast-moving animals. The results obtained with this method correlate well (Rs = 0.76), with the activity index determined by a visual *detailed method*. The automatic analysis of animal behaviour described above is many times faster than visual analysis and enables the precise assessment of the daily activity of fast-moving groups (herds) of animals. The automatic method of activity assessment for very fast-moving animals along with the programming tools enable on-line image analysis, even by a computer with average parameters in terms of computational efficiency (please see the chapter Materials and Methods for more information). In addition, the image processing method presented above has proven itself on footage from a security camera with average technical parameters. The use of the system described in this paper makes it possible to obtain an activity index with a sub-second resolution, which allows it to be used in on-line mode as a detector of abnormal animal activity, e.g. early detection of illnesses or sudden events that manifest themselves as increased or decreased activity compared to the standard activity profile. This method can be applied to other species of animals whose activity is equally difficult to assess visually.

## Data Availability

All data generated or analysed during this study are included in this published article. All materials are housed at the Department of Experimental Computer Physics, Institute of Physics, Jagiellonian University Cracow, Poland. The datasets used and/or analysed during the current study are available from the corresponding author upon request.
