# Author Correction: A novel method for automatically analysing the activity of fast-moving animals: a case study of *Callimico goeldii* monkeys housed in a zoological garden

**DOI:** 10.1038/s41598-023-40101-z

**Published:** 2023-08-08

**Authors:** Zenon Nieckarz, Jacek Nowicki, Karolina Labocha, Krzysztof Pawlak

**Affiliations:** 1 Department of Experimental Computer Physics, Institute of Physics, Jagiellonian University in Cracow, Cracow, Poland; 2 Department of Genetics, Animal Breeding and Ethology, University of Agriculture in Cracow, Cracow, Poland; 3 Silesian Zoological Garden, Chorzów, Poland; 4 Department of Zoology and Animal Welfare, University of Agriculture in Cracow, Aleja Adama Mickiewicza 24/28, 30‑059 Kraków, Poland

Correction to: *Scientific Reports* https://doi.org/10.1038/s41598-023-38472-4, published online 16 July 2023

The original version of this Article contained errors in the names of the authors Zenon Nieckarz, Jacek Nowicki, Karolina Labocha & Krzysztof Pawlak, which were incorrectly given as Nieckarz Zenon, Nowicki Jacek, Labocha Karolina & Pawlak Krzysztof.

The original Article has been corrected.

